# Prospective study of stress, depression and personality in myasthenia gravis relapses

**DOI:** 10.1186/s12883-020-01802-4

**Published:** 2020-06-29

**Authors:** Anca Bogdan, Carolina Barnett, Abdulrahman Ali, Mohammed AlQwaifly, Alon Abraham, Shabber Mannan, Eduardo Ng, Vera Bril

**Affiliations:** 1 Ellen and Martin Prosserman Centre for Neuromuscular Diseases, Division of Neurology, Department of Medicine, Toronto General Hospital, University Health Network, University of Toronto, 5EC, Room 309 200 Elizabeth St, Toronto, ON M5G 2G4 Canada; 2 Institute of Health Policy, Management and Evaluation, Dalla Lana School of Public Health, University of Toronto, Toronto, Canada; 3 National Neuroscience Institute, King Fahad Medical City, Riyadh, Saudi Arabia; 4 Qassim University, Buraydah, Al-Qassim Saudi Arabia; 5 Neuromuscular Diseases Unit of the Department of Neurology, Tel Aviv Sourasky Medical Center, The Sackler Faculty of Medicine, Tel Aviv University, Tel Aviv, Israel; 6 Institute for Research and Medical Consultations, Imam Abdulrahman Bin Faisal University, Dammam, Saudi Arabia

**Keywords:** Myasthenia gravis, Relapse, Trigger factors, Stress, Depression, Personality

## Abstract

**Background:**

Psychopathology and personality traits may influence the course of autoimmune disorders. With this prospective longitudinal cohort study, we aimed to assess personality, stress and depression in myasthenia patients who relapse and those who remain stable or improve (non-relapsers).

**Method:**

We collected data from 155 consecutive adult patients with confirmed MG attending the Neuromuscular Clinic, Toronto General Hospital, between March 2017 and July 2018, for this study. Patients were assessed at baseline and 6 months, or at the time of MG relapse. At both visits, the patients were assessed clinically and were asked to complete self-administered questionnaires for disease severity, chronic stress and depression. Personality type was assessed at baseline only. Relapsing patients were defined as those patients with MGII score increasing by more than 5.5 points from visit 1 to visit 2.

**Results:**

Relapsers had higher baseline scores for depression (*p* = 0.01) and the change in disease severity correlated with the change in depression score (*r* = 0.2534, *p* = 0.0015, 95% CI: 0.098 0.3961). Higher levels of stress at baseline and neuroticism predicted higher relapse rates (*p* = 0.01 and *p* < .0001, respectively). In the linear regression model, with change of the MGII score as the dependent variable, change in depression scores (*p* = 0.0004) and age (*p* = 0.03) predicted change in disease severity.

**Conclusions:**

Since emotional factors and personality type may influence MG, attention to these factors might improve care in MG patients.

## Background

Acquired myasthenia gravis (MG) is a chronic autoimmune disorder caused by an antibody-mediated impairment of neuromuscular transmission resulting in fluctuating muscle weakness [1]. The prevalence of the disease is estimated to be 10 to 20 cases per 100,000 population [2].

In addition to infection and medication, which are common triggers of MG exacerbations, psychopathology, personality and coping mechanisms may influence the course of the disease [3]. Maladaptive traits such as neuroticism are associated with passive copings and cause high-stress levels [4]. Severe and prolonged mental stress and emotional arousal can affect immune function and may lead to onset or relapses of MG [5]. Also, MG is an unpredictable disease with a relapsing and remitting course necessitating the use of chronic medication with potential side-effects that impair quality of life [6–8] and may cause psychological stress and predispose to depression [9]. Patients with MG likely experience chronic dysregulation of the hormonal stress axis and the immune system, aggravating the disease itself but also leading to secondary psychopathological abnormalities. In a recent cross-sectional study, 17.3% of MG patients had depression, [10] although a wide range of depression rates among MG patients has been described previously [11–17].

We aimed to assess personality, stress and depression factors in MG patients with relapses compared with those who did not relapse in a prospective longitudinal cohort study hypothesizing a relationship between personality type, chronic stress, and the likelihood of relapse.

## Methods

### Participants

We invited all consecutive adult patients with confirmed MG attending the Prosserman Family Neuromuscular Clinic, Toronto General Hospital, between March 2017 and July 2018, to participate in this study. Patients were eligible if they were over 18 years old and able to understand the study procedures. The diagnosis of MG was confirmed by a neuromuscular physician (VB, CB) based on the clinical presentation, abnormal single fiber electromyography studies, and positive antibody titres, if available.

### Ethics approval and consent to participate

The University Health Network Research Ethics Board approved the study and all patients provided written informed consent.

### Measures

Patients were assessed at baseline and 6 months, or earlier if there was a relapse of MG. At both visits, the patients were assessed clinically and were asked to complete the Myasthenia Gravis Impairment Index (MGII) for disease severity, [18–20] the short version of the Trier Inventory for Assessment of Chronic Stress (TICS) [21, 22] and Beck’s Depression Inventory – Second Edition (BDI-II) [23]. Additionally, patients completed the Big 5 Personality Inventory (NEO-PI-Revised) [24] at baseline only. Information on background medical and mood disorders was also recorded.

Patients were categorized as non-relapsers (stable/better) or relapsers (relapse/worsening) using the MGII score values from baseline to completion visits. Relapsers were defined as those patients with MGII score increasing by more than 5.5 points from visit 1 to visit 2 [20].

### Statistical analysis

Analyses were performed using R, version 3.5.0. Results are presented as counts (N) and proportions (%) or means +/− standard deviations as appropriate. We compared clinical and demographic variables in relapsers and non-relapsers. We calculated and compared the rate of relapse for patients with and without higher levels of stress and depression. For continuous variables, the differences between groups were analyzed with t-tests and we used chi-square tests to compare proportions. We used Pearson correlation coefficients to evaluate the correlation of disease severity with changes in stress level and depression. We built a multivariable linear regression model using the change in MGII as the dependent variable to find the association between change in disease severity, level of stress, depression and personality type. We also incorporated relevant demographic and clinical factors (age, sex, thymoma, MG type, change in medications, MGII at baseline) in the models. We retained the best fitting model, and reported the estimates and standard error for each variable. *P*-values < 0.05 were considered statistically significant for all analyses.

## Results

A total of 179 patients entered the study and completed the first visit, and 155 patients returned all questionnaires after the second visit. Reasons patients were not enrolled included those who: were acutely ill, declined research, lived at a distance and would not return, had language barriers, or were senile. Of those completing the study, 51.6% were women. Age ranged from 22 to 85 years and MG duration from 1 to 46 years with a mean duration of 11.3 ± 9.1 years. Mean MGII (disease severity) was 14.0 after the first visit and 15.3 after the second visit; 82.6% of patients had generalized disease, and the others had ocular disease; about one quarter were diagnosed with thymoma and 56.1% had a thymectomy; 111 of 155 patients had antibody tests and AChR antibodies were found in 57.5% and anti-MuSK antibodies in 5.4%. The demographics, personality type, and prevalence of stress and depression in the cohort at baseline have been described previously [10]. At baseline, 17.3% had depression (BDI-II ≥17) and 11.7% had higher levels of stress (TICS ≥60).

Of 155 patients, 33 (21.3%) were relapsers; of the remaining 122 patients (non-relapsers), 19 (12.3%) were better and 103 (66.5%) were stable. At baseline, 6% of non-relapsers, and 9% of relapsers had a diagnosed mood disorder: depression, anxiety, panic attacks.

Patients who had a relapse also had worsening in the BDI-II scores, but no significant change on the TICS scale. The proportion of MG patients with symptoms of depression at the end of the study was 27.3% among relapsers and 12.5% for non-relapsers (*p* = 0.01). Overall, comparing patients with (BDI-II ≥ 17) and without (BDI-II < 17) depression at baseline, we found significant differences with regards to their age at onset (39.2 vs 49.4 years, *p* = 0.003), age at the time of the study (50.5 vs 60.0 years, *p* = 0.002) and associated levels of stress—TICS scores (61.2 vs 29.6, *p* < .0001). They had similar disease duration (11.4 vs 10.6 years) and genders were almost equally distributed (54.2% females and 45.8% males). There were no significant baseline differences in the proportion of relapsers and non-relapsers receiving immunosuppressant medications. Patients with a relapse had a slightly higher, but insignificant, mean daily dose of prednisone (17.2 ± 13 vs 12.8 ± 8, *p* = 0.15). Those who relapsed had more severe disease as measured by the MGII at baseline. Demographics of our patient cohort and clinical findings are shown in Table 1.

**Table 1:** Comparison between MG relapsers and non-relapsers

Variable	Total (*n* = 155)	Non-relapsers (*n* = 122)	Relapsers (*n* = 33)	*p* value
Age (y)	58.5 ± 14.0	58.5 ± 14.5	58.7 ± 12.3	0.9
Sex F	80 (51.6)	64 (52.5)	16 (48.5)	0.7
Disease duration (y)	10.7 ± 8.8	10.7 ± 8.6	10.8 ± 9.6	1.0
Age of onset (y)	47.8 ± 15.7	47.8 ± 16.2	47.9 ± 14.1	1.0
Generalized	128 (82.6)	97 (79.5)	31 (93.9)	0.05
MGII Score	1.4 ± 8.4	−1.6 ± 5.3	12.2 ± 7.7	**< 0.0001**
Thymoma	42 (27.1)	33 (27.0)	9 (27.3)	1.0
Thymectomy	87 (56.1)	67 (54.9)	20 (60.0)	0.6
AchRAb	64 (57.7)	53 (60.2)	11 (47.8)	0.2
MuSK Ab	6 (5.4)	4 (4.5)	2 (8.7)	0.5
TICS-S	−5.8 ± 16.8	−6.8 ± 16.4	−1.8 ± 17.9	0.12
BDI-II	−0.4 ± 5.3	−1.0 ± 5.2	1.7 ± 5.5	**0.01**
Potential triggers	67 (43.2)	52 (42.6)	15 (45.5)	0.8
Personal problems	26 (16.7)	17 (13.9)	9 (27.3)	0.07
Other illnesses	48 (31.0)	35 (28.7)	13 (39.4)	0.2
**Medications (baseline)**				
Prednisone	97 (63)	75 (61)	22 (67)	0.59
Prednisone dose/day (mg)	13.8 ± 9.7	12.7 ± 8.3	17.2 ± 13.2	0.15
Azathioprine	56 (41)	48 (40)	8 (24)	0.10
Mycophenolate	26 (17)	22 (18)	4 (12)	0.40
**Reduction in Immunosuppressant (baseline to follow up)**	48 (31)	40 (33)	8 (24)	0.32

Values are means ± SD or n (%)

Statistics are calculated using unpaired two-tailed T-test for numerical data and Fisher/Chi-Square test for categorical values

For MGII, TICS and BDI, values are change scores from the baseline visit

Potential triggers: infections, medication change, hospital admissions

CI for difference in prednisone dose: [−0.2, 0.089.1] mg (crosses 0)

Baseline stress was associated with relapse rate: in patients with baseline TICS < 30 (“rarely” had a stressful event), the relapse rate was 13.9%, increasing to 22.6% with TICS between 30 and 59 (“sometimes” had a stressful event) and 31.6% with TICS score ≥60 (“often” had a stressful event) (*p* value = 0.01, chi-square test). For those without depression at baseline (BDI 0–10) the relapse rate was 17.6%, with mild mood disturbance (BDI 11–16) 33.3% and with greater depression (BDI ≥ 17) 24.1% (p value = 0.04, chi-square test). The change in disease severity had a weak correlation with change in depression score (r = 0.2534, *p* = 0.0015, 95% CI: 0.098 0.3961) but no correlation with change in chronic stress score.

Table 2 shows disease severity, stress, depression and relapse rate across personality types at baseline. No extroverted patients were found in this cohort. Significantly higher stress levels were observed for neuroticism and openness, and the highest relapse rate was associated with neuroticism although the patient numbers are small in this cohort. There were no important differences with regards to presence of infections, admissions, change in medication or occurrence of personal problems across personality types, but comorbidities were more frequent in the agreeableness group than the openness group (58.3% versus 11.1%, *p* = 0.03).

**Table 2:** Personality, disease severity, stress, depression and relapse rates in 155 MG patients

Personality	% of total	MGII	∆MGII	TICS	∆TICS	BDI-II	∆BDI-II	Relapse Rate (%)
Agreeableness	33	14.3	2.7	37.2	−4.6	9.9	−0.9	23.5
Conscientiousness	30	12.5	0.8	37.3	−8.6	0.21	−0.1	21.7
Neuroticism	3	21.8	6	48.5	−15	11.5	−5.3	50
Openness	34	14.5	0.3	45.9	−3.7	11.1	0.1	17
p-value		0.6	0.32	0.03	0.31	0.08	0.22	**< 0.0001**

Categorial variables compared by chi-squared test and continuous variables by **ΑΝΟVΑ **

% of total is % of total cohort

In the linear regression model using change of the MGII score as the dependent variable, the following variables were included: age, sex, change in TICS score, change BDI-II score, personality type, disease duration, acute infections, change in medical treatment, personal problems, comorbidities and baseline MGII score. The final model had R^2^:0.15 and Likelihood Ratio = 25.11, with *p* = 0.0006. The only variables predicting change in disease severity were change in depression scores (*p* = 0.0004) and age (*p* = 0.03). Model estimates are found in Table 3.

**Table 3:** Linear regression model with change in MGII as the dependent variable

	Coefficient	SE	t	Pr(>t)
Intercept	−4.542	3.473	−1.31	0.193
Age	0.104	0.049	2.09	**0.038**
Sex, Male	1.251	1.386	0.90	0.368
Disease Duration	0.020	0.076	0.26	0.793
Personality C	−1.750	1.723	−1.02	0.311
Personality N	5.990	4.301	1.39	0.161
Personality O	−3.148	1.697	−1.86	0.065
∆TICS	0.016	0.041	0.42	0.678
∆BDI-II	0.450	0.126	3.57	**< 0.001**
Medical Y	1.776	1.357	1.31	0.193
Personal Y	2.385	1.895	1.26	0.210
Comorbidities Y	−1.383	1.250	−0.92	0.360
Baseline MGII	−0.083	0.050	−1.674	0.096

Only change in depression and age predicted disease severity in this patient cohort

*SE* standard error

∆ = change

## Discussion

In this prospective, longitudinal cohort study, we found a positive association between MG relapses and depression. Baseline stress levels also predicted relapses. When adjusting for confounders, change in depression and age were significantly associated with relapses, although baseline severity on MGII was not a predictor in this study. A one-point increase in MGII score was associated with a 0.5 point increase in BDI-II score.

Our study has shown a higher prevalence of depression at 17% compared to the Canadian and global estimates in the adult population of 5.4 and 4.4% [25, 26]. Our increased rate is similar to previous reports of more prevalent depression and other psychiatric issues in MG patients [13, 16, 17]. Interestingly, our MG patients with depression were younger, with an earlier disease onset and they had higher scores of associated stress and disease severity, likely due to different methods of assessing depression in different studies or different study cohorts.

We found that the rate of relapse increased with increasing stress scores at baseline. However, this finding is confounded by the correlation between disease severity at baseline and depression. Because patients who relapsed had higher disease severity scores at baseline, we cannot assess if high depression scores are triggering the flare-ups in this cohort. Previously it has been reported that onset of MG is triggered by physical/emotional stress in up to 20% of patients [5] and this is comparable to the 14.8% of patients in our cohort who reported a stressful event at onset of MG. Many patients report exacerbation of symptoms after experiencing mental stress (60.6%), [5] but in our cohort, the relapse rate of 31.6% in those having a frequent stressor was lower and this finding might be related to the different MG populations studied as our patients were generally less affected out-patients.

We found a positive correlation of depression and disease severity. Others have reported that disease severity and stressful life events were associated with depressive symptoms [4] and poor quality of life correlated with symptoms of depression [5–7]. Also, it has been reported that depression in MG is associated with early stage of disease, lack of response to treatment, and use of corticosteroids [12]. Most of our patients were on chronic steroid therapy perhaps accounting for the patients’ depressive state. Alterations in corticosteroid and/or catecholamine level in response to stressors may play a key role in the development of MG exacerbation [27] and flares in other autoimmune disorders [28]. Only about 15% of our patients recognized a stressful event related to family, work or personal injuries associated with the onset of the disease, but this result may be limited by recall bias. Other retrospective studies report that up to 80% of patients experience uncommon emotional stress before disease onset although this seems high given our results. However, not only does stress cause disease, but the disease itself also causes significant stress in the patients, creating a vicious cycle [29]. Therefore, it is very difficult to establish causal pathways between stress and MG severity.

The rate of relapse was 21.3% in our patient cohort and the overall disease severity was low as shown by the MGII values. Other studies have reported relapse rates up to 34%, [30] MG crisis in 20–30%, [31, 32] or even lower [33]. It is likely that the brief duration of follow-up of 6 months prevented observation of a higher relapse rate in our study cohort. Longer studies may be needed to further study this question. In our study, stress level and relapse rate differed across personality types. Neuroticism was associated with the highest scores of stress, depression, disease severity and relapse rate in our study, similar to previous findings, [4] but since only a few patients had neuroticism, our findings must be considered with caution.

A surprising finding in our study was the high score of depression and stress associated with openness, but this group of mostly men had the lowest relapse rate. An explanation may be that they had better coping mechanisms using a more problem-focused method of handling stressful experiences, [34] and having more emotional inhibition [35]. Research findings show positive relationships between openness and active coping and positive reinterpretation [36]. We found that the lowest rates of disease severity and depression were associated with conscientiousness. Previously, it was reported that adaptive personality traits (e.g., high extraversion and conscientiousness) were less affected by daily stresses, although other studies differ [4, 37].

Some limitations of our study are inclusion of patients with established MG who are followed in our clinic (and so being unable to determine if depression and stress preceded the diagnosis or developed later), the short duration of follow-up, the absence of socioeconomic data, the lack of formal psychiatric evaluation, potential confounding of MG symptoms by those with affective disorders, inclusion of relatively stable outpatients with limited disease severity and uncertainty about the temporal association of depression and MG triggers or relapses.

The strengths of our study are the prospective, longitudinal study design, the large number of patients recruited, the use of a validated measure of disease severity recognized scales for assessing depression and stress, and a less severely affected MG outpatient cohort that avoids overestimation of depression.

In summary, we found that baseline stress levels and increasing depression were associated with higher relapse rates in MG but that personality type did not clearly influence relapse rate.

## Conclusion

Since emotional factors and personality type may influence MG, attention to these factors might improve care in MG patients.

## Data Availability

Individual de-identified participant data and related documents such as study protocol and statistical analysis plan will be shared with qualified investigators for a period of 12 months after publication of this paper, pending review and approval of all detailed requests by the UHN Research Ethics Board. All such requests should be directed to the corresponding author.

## Abbreviations

MG: Myasthenia gravis
MGII: Myasthenia Gravis Impairment Index
TICS: Trier Inventory for Assessment of Chronic Stress, short version
BDI-II: Beck's Depression Inventory – Second Edition
NEO-PI-Revised: Big 5 Personality Inventory
AChR: Acetylcholine receptor
Anti-MuSK: Anti muscle specific kinase
CI: Confidence interval
